# CDH1 regulates E2F1 degradation in response to differentiation signals in keratinocytes

**DOI:** 10.18632/oncotarget.13636

**Published:** 2016-11-26

**Authors:** Randeep K. Singh, Lina Dagnino

**Affiliations:** ^1^ Department of Physiology and Pharmacology, Children's Health Research Institute and Lawson Health Research Institute, The University of Western Ontario, London, Ontario N6A 5C1, Canada

**Keywords:** keratinocytes, epidermis, E2F1

## Abstract

The E2F1 transcription factor plays key roles in skin homeostasis. In the epidermis, E2F1 expression is essential for normal proliferation of undifferentiated keratinocytes, regeneration after injury and DNA repair following UV radiation-induced photodamage. Abnormal E2F1 expression promotes nonmelanoma skin carcinoma. In addition, E2F1 must be downregulated for proper keratinocyte differentiation, but the relevant mechanisms involved remain poorly understood. We show that differentiation signals induce a series of post-translational modifications in E2F1 that are jointly required for its downregulation. Analysis of the structural determinants that govern these processes revealed a central role for S403 and T433. In particular, substitution of these two amino acid residues with non-phosphorylatable alanine (E2F1 ST/A) interferes with E2F1 nuclear export, K11- and K48-linked polyubiquitylation and degradation in differentiated keratinocytes. In contrast, replacement of S403 and T433 with phosphomimetic aspartic acid to generate a pseudophosphorylated E2F1 mutant protein (E2F1 ST/D) generates a protein that is regulated in a manner indistinguishable from that of wild type E2F1. Cdh1 is an activating cofactor that interacts with the anaphase-promoting complex/cyclosome (APC/C) ubiquitin E3 ligase, promoting proteasomal degradation of various substrates. We found that Cdh1 associates with E2F1 in keratinocytes. Inhibition or RNAi-mediated silencing of Cdh1 prevents E2F1 degradation in response to differentiation signals. Our results reveal novel regulatory mechanisms that jointly modulate post-translational modifications and downregulation of E2F1, which are necessary for proper epidermal keratinocyte differentiation.

## INTRODUCTION

The E2F family of transcription factors plays important roles in myriad physiological processes. The more widely characterized functions of this family include their regulatory roles in cell cycle progression and tumourigenesis, although it is now well established that E2F proteins also modulate such diverse processes as metabolism, apoptosis, and differentiation [1, 2]. In mammals, eight E2F and three DP proteins have been described [3], but the elucidation of their specific functions has been complicated in part due to their functional redundance under certain conditions.

E2F1 was the first member of this family identified, and is ubiquitously expressed, although its abundance is tightly regulated in tissues composed of undifferentiated and differentiated cell populations, such as the epidermis and the central nervous system [4–6]. For example, E2F1 is more abundant in keratinocyte stem cells and their undifferentiated, transit amplifying progeny than in terminally differentiated cells. Overexpression of E2F1 causes nonmelanoma skin carcinoma. Conversely, keratinocyte differentiation requires E2F1 downregulation, which occurs through transcriptional and post-translational mechanisms [7]. Physiological activation of keratinocytes to regenerate the wounded epidermis is associated with E2F1 upregulation, and is severely impaired in the absence of this transcription factor [8]. Due to their chronic exposure to solar UV radiation, epidermal keratinocytes have developed efficient mechanisms for DNA repair to avoid carcinogenic transformation, which rely on E2F1 and various other factors [9, 10].

The importance of post-translational modifications for E2F1-regulated functional outcomes has been extensively studied in the context of responses to DNA damage in normal epidermal keratinocytes and various tumour cell types. Several studies have identified serine phosphorylation, arginine methylation and lysine acetylation in E2F1 as key events triggered by formation of DNA strand breaks [1, 10]. Less well understood are the pathways and mechanisms that regulate E2F1 post-translationally during physiological events, such as keratinocyte differentiation. In the epidermis, keratinocytes are induced to differentiate in response to several stimuli, which include Ca^2+^-activated pathways and Notch signalling [7, 11]. These events can be recapitulated in cultured primary keratinocytes by elevating the extracellular Ca^2+^ concentration from ~0.05 to ≥ 0.1 mM. Under these conditions, E2F1 undergoes p38β-dependent phosphorylation at S403 and T433 and ubiquitylation, culminating with its degradation [7, 12, 13]. It is well established that E2F1 degradation is essential for proper keratinocyte differentiation [7, 12, 13]. However, the detailed molecular mechanisms that modulate E2F1 levels during differentiation have not been explored. We now show a specific, unique role of residues S403 and T433 in modulating E2F1 changes in subcellular localization, as well as K11- and K48-linked ubiquitylation, necessary for proper keratinocyte differentiation in response to Ca^2+^ stimulation.

## RESULTS

### Distinct molecular determinants of E2F1 stability during differentiation and DNA damage

E2F1 has a relatively short half-life that ranges between 1 h and 4 h in most cell types examined to-date, and changes in its stability can rapidly occur to allow cell responses to various stresses. For example, E2F1 is stabilized in response to DNA damage caused pharmacologically, by UV or ionizing radiation. This phenomenon has been widely characterized, and the role of several key E2F1 residues implicated in stabilization is well established [1]. However, whether those same residues are also involved in regulating E2F1 turnover during terminal differentiation is not known. We addressed this question in primary epidermal keratinocytes, in which E2F1 is rapidly degraded upon induction of differentiation [7].

**Figure 1:**
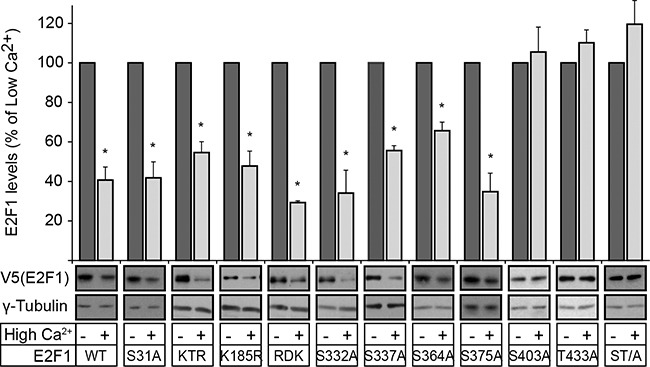
S403 and T433 mediate E2F1 degradation during keratinocyte differentiation Vectors encoding V5-tagged wild type (WT) or the indicated E2F1 mutants were transfected in undifferentiated keratinocytes. Twenty-four hours after transfection, cells were treated with cycloheximide (CHX, 100 μg/ml), and 30 min later the culture medium was replaced with Low Ca^2+^ or High Ca^2+^ medium supplemented with CHX. Cell lysates were prepared 2.5 h later and analyzed by immunoblot for the indicated proteins, using anti-V5 antibodies, or γ-tubulin, as loading control. The histograms represent normalized densitometric quantification of each E2F1 protein (mean + SEM), and are expressed as the percentage of a given E2F1 form relative to its abundance in Low Ca^2+^ medium, which is set at 100%. The asterisks indicate P<0.05 (ANOVA).

DNA damage in epidermal keratinocytes and other cell types induces ATM and/or ATR-mediated phosphorylation of S31, as well as Chk2-mediated phosphorylation of S364 in human E2F1, resulting in increased stability [14, 15]. Phosphorylated S31 binds to 14-3-3-τ, which in turn decreases E2F1 ubiquitylation and degradation [15]. To determine the role of these two residues during differentiation, we exogenously expressed wild type, S31A or S364A E2F1 in primary mouse keratinocytes, and assessed their abundance 24 h after induction of differentiation by culture in medium containing 1.0 mM Ca^2+^ (“High Ca^2+^ medium”). Both mutant and wild type E2F1 showed similar decreases of 45-60% in response to Ca^2+^ (Figure 1).

Several E2F1 lysine residues can be acetylated, methylated and/or neddylated. Whereas acetylation of K117, K120 and K125 upon DNA damage increases E2F1 stability [16], their neddylation increases E2F1 degradation in the absence of DNA damage [17]. However, we observed that mutation of these three lysines to arginine to produce the mutant E2F1 “KTR” did not alter degradation during differentiation (Figure 1). Similar susceptibility to differentiation-induced degradation was observed in a mutant E2F1 K185R (Figure 1), in which methylation and neddylation, known to regulate stability, are precluded [17].

Methylation of R111 and R113 modulates the pro-apoptotic responses to E2F1, and Arg-to-Lys mutation of these residues reportedly yields a mutant “E2F1 RDK” protein with increased stability in osteosarcoma cells [18]. We observed that the levels of this mutant also decreased by about 70% upon differentiation (Figure 1). Finally, although phosphorylation of S332, S337 and S375 is important for E2F1 association with the retinoblastoma protein, transcriptional activity and protection from degradation in tumour cell lines, these residues are also dispensable for E2F1 degradation in differentiating keratinocytes (Figure 1). Our studies also confirmed the previously reported essential role of S403 and T433 in mediating E2F1 degradation during differentiation [7], as mutation of one or both of these residues to alanine yielded E2F1 proteins resistant to Ca^2+^ -induced degradation (Figure 1). We similarly examined the changes in steady-state levels of wild type and mutant E2F1 proteins following DNA damage induced by etoposide (Supplementary Figure S1). Etoposide treatment increased the abundance of wild type, K185R and RDK E2F1 proteins, irrespective of the differentiation status of the keratinocytes. In contrast, the abundance of E2F1 S31A remained unaltered in etoposide-treated cells, indicating that S31 is essential for a normal response to etoposide damage in both differentiated and undifferentiated cells. Significantly, etoposide also increased the abundance of the double S403A/T433A E2F1 mutant (hereafter termed E2F1 ST/A), consistent with the notion that S403 and T433 are not required for E2F1 stabilization upon DNA damage. Together, our studies indicate that the molecular determinants that regulate E2F1 abundance during epidermal differentiation are distinct from those activated during DNA damage.

### Role of nuclear export in differentiation-induced E2F1 degradation

**Figure 2:**
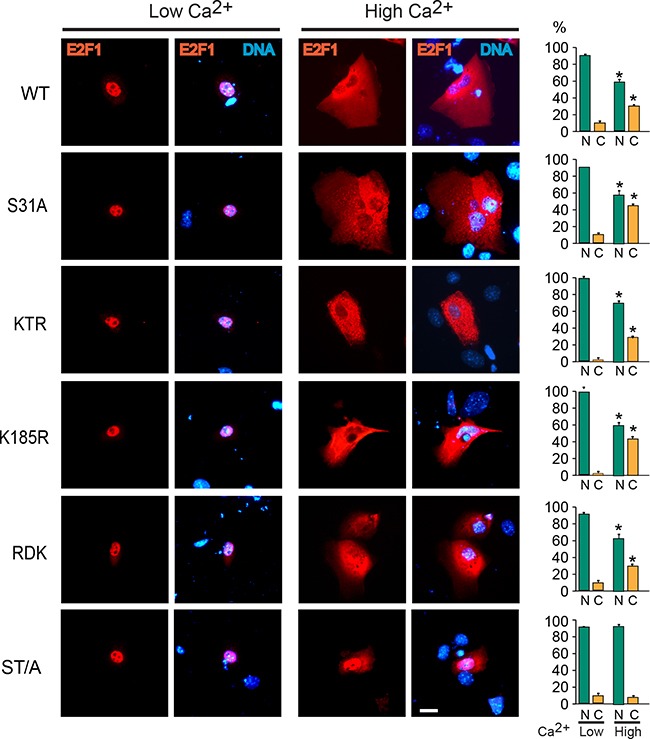
S403 and T433 are required for E2F1 nuclear export during keratinocyte differentiation Undifferentiated keratinocytes were transfected with vectors encoding V5-tagged wild type (WT) or the indicated E2F1 mutant proteins. Four hours after transfection, the culture medium was replaced with Low Ca^2+^ or High Ca^2+^ medium, and the cells were processed for immunofluorescence microscopy 24 h later, using anti-V5 antibodies. DNA was visualized with Hoechst 33342. The values in the histograms represent the percentage of cells (mean + SEM, n=3) that exhibited nuclear (N) or cytoplasmic (C) E2F1 distribution. The asterisks indicate P<0.05 relative to values in the corresponding subcellular compartments in Low Ca^2+^ medium (ANOVA). Bar, 16 μm.

**Figure 3:**
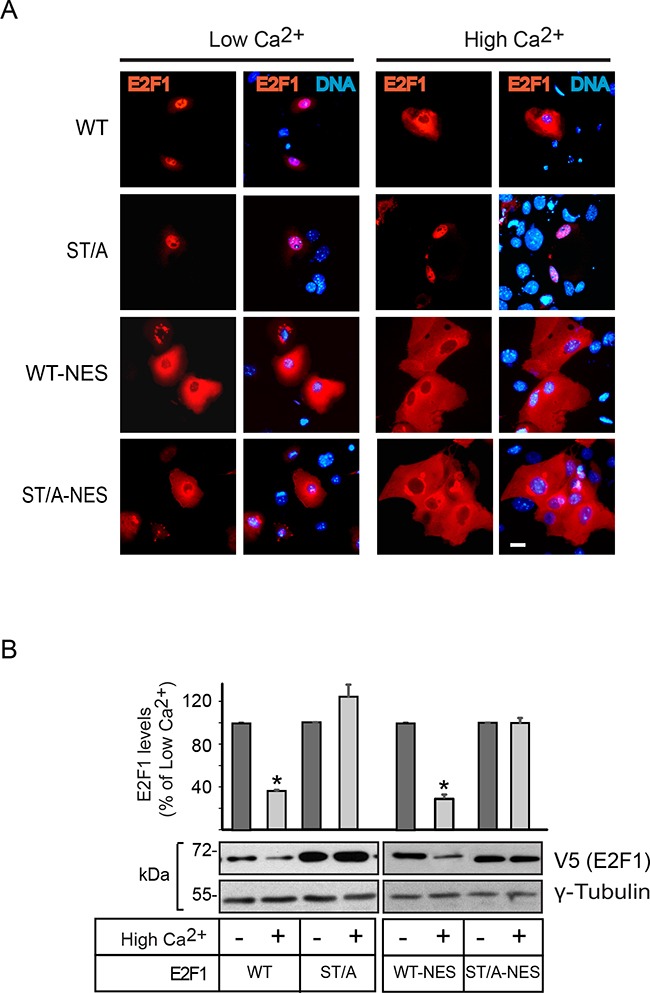
Effect of constitutive nuclear export on E2F1 abundance in differentiating keratinocytes **A**. Undifferentiated keratinocytes were transfected with vectors encoding V5-tagged wild type (WT) or the indicated E2F1 mutant proteins. Four hours after transfection, the culture medium was replaced with Low Ca^2+^ or High Ca^2+^ medium, and the cells were processed for immunofluorescence microscopy 24 h later, using anti-V5 antibodies. DNA was visualized with Hoechst 33342. **B**. Cells transfected as described in (A) were cultured for 24 h in Low Ca^2+^ medium. Cycloheximide was added (100 μg/ml), and 30 min later the cells were cultured for 2.5 h in Low Ca^2+^ or High Ca^2+^ medium supplemented with cycloheximide. Cell lysates were prepared and analyzed by immunoblot with antibodies against V5 or γ-tubulin, used as loading control. The histograms represent normalized densitometric quantification of each E2F1 protein (mean + SEM, n=3), and are expressed as the percentage of a given E2F1 form relative to its abundance in Low Ca^2+^ medium, which is set at 100%. The asterisks indicate P<0.05 (ANOVA).

We have previously shown that E2F1 undergoes nucleocytoplasmic shuttling, and that it exhibits a net nuclear CRM1-dependent export into the cytoplasm in differentiating keratinocytes. The latter is necessary for degradation, as leptomycin B inhibition of CRM1 results in E2F1 stabilization and constitutive nuclear localization [12, 19]. Consistent with this notion, analyses of the subcellular distribution of the E2F1 mutants described above, and which exhibited differentiation-induced degradation, revealed nuclear export that was indistinguishable from that observed with the wild type protein (Figure 2 and Supplementary Figure S2). In contrast, the single S403A and T433A E2F1 forms, as well as E2F1 ST/A, remained nuclear in differentiated cells (Figure 2, Supplementary Figure S2) Given the complexity of E2F1 regulatory mechanisms, we wished to further investigate if nuclear export was the principal pathway involved in its Ca^2+^-induced degradation. To this end, we generated wild type and ST/A mutants that also contained the leucine-rich consensus nuclear export sequence (NES) from the simian virus 40 large T antigen (LPPLERLTL) at the C-terminus. Addition of this NES to both E2F1 forms yielded proteins that were constitutively cytoplasmic, irrespective of the differentiation status of the keratinocytes (Figure 3A). However, whereas E2F1-NES exhibited a marked decrease in abundance in response to Ca^2+^, the levels of E2F1-ST/A-NES remained unaltered (Figure 3B). Thus, these observations demonstrate that nuclear export depends on intact S403 and T433 residues, but that it is not sufficient to promote E2F1 degradation in response to differentiation in keratinocytes.

### Potential role of S403 and T433 phosphorylation in regulation of E2F1 subcellular localization

**Figure 4:**
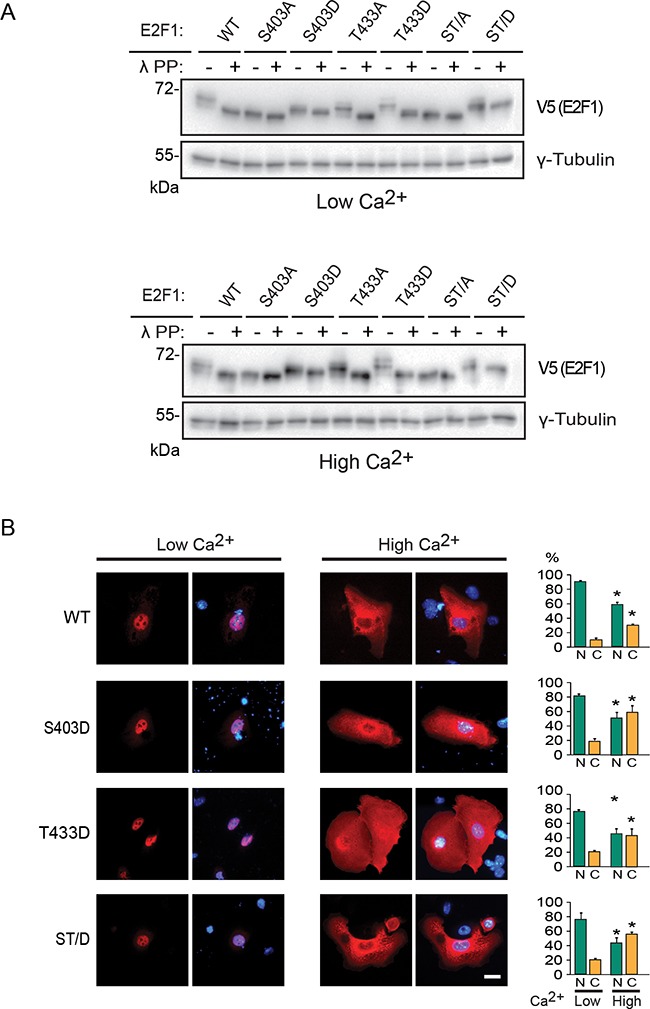
Characterization of pseudophosphorylated E2F1 mutant proteins **A**. Undifferentiated keratinocytes were transfected with vectors encoding V5-tagged wild type (WT) or the indicated E2F1 mutant proteins. Four hours after transfection, the culture medium was replaced with Low Ca^2+^ or High Ca^2+^ medium. After 24 h, cell lysates were prepared and incubated in the presence or absence of λ phosphatase (λ PP; 4 U/mg of lysate protein), resolved on denaturing polyacrylamide gels, and analyzed by immunoblot, with antibodies against V5 or γ-tubulin, used as loading control. The results shown are representative of three experiments. **B**. Cells transfected as described in (A) were processed for immunofluorescence microscopy following 24 h of incubation in Low Ca^2+^ or High Ca^2+^ medium, using anti-V5 antibodies. DNA was visualized with Hoechst 33342. The values in the histograms represent the percentage of cells (mean + SEM, n=3) that exhibited nuclear (N) or cytoplasmic (C) E2F1 distribution. The asterisks indicate P<0.05 relative to values in the corresponding subcellular compartments in Low Ca^2+^ medium (ANOVA). Bar, 16 μm.

The above observations prompted us to examine if phosphorylation of S403 and/or T433 might play additional roles in E2F1 turnover. To address this possibility, we generated pseudophosphorylated E2F1 mutant forms, in which S403, T433 or both were substituted with aspartic acid. We initially compared the mobility of these mutant proteins with that of wild type E2F1 on denaturing polyacrylamide gels. Several wild type E2F1 species were evident in lysates from both differentiated and undifferentiated keratinocytes, which collapsed into a single band upon treatment with λ phosphatase, indicating that E2F1 is phosphorylated at one or more residues in these cells (Figure 4A). In undifferentiated cells, substitution of S403 by either alanine or aspartic acid gave rise to mutant E2F1 proteins that migrated with similar mobility to that of unphosphorylated wild type E2F1. Treatment with λ phosphatase did not appreciably alter the mobility of E2F1 S403A and S403D (Figure 4A), indicating that S403 is likely phosphorylated in these cells. Substitution of T433 by alanine or aspartic acid gave rise to an E2F1 form with intermediate mobility between the wild type and the S403 mutant, and which further collapsed to a single species in the presence of λ phosphatase (Figure 4A). λ phosphatase treatment had no effect on the mobility of E2F1 ST/A or ST/D. However, the latter exhibited lower mobility than the former, likely related to the overall charge of the protein under these conditions (Figure 4A). Similar results were obtained using lysates prepared from differentiated keratinocytes in the presence and absence of λ phosphatase (Figure 4A). Next, we examined if E2F1 pseudophosphorylation is sufficient to mediate nuclear export in response to Ca^2+^. We observed that substitution of S403, T433 or both by aspartic acid produced mutant proteins predominantly localized to the nucleus in undifferentiated cells, and which were efficiently redistributed to the cytoplasm upon differentiation in a manner that was indistinguishable from that of wild type E2F1 (Figure 4B).

### Differentiation-specific role of S403 and T433 regulation of E2F1 stability

**Figure 5:**
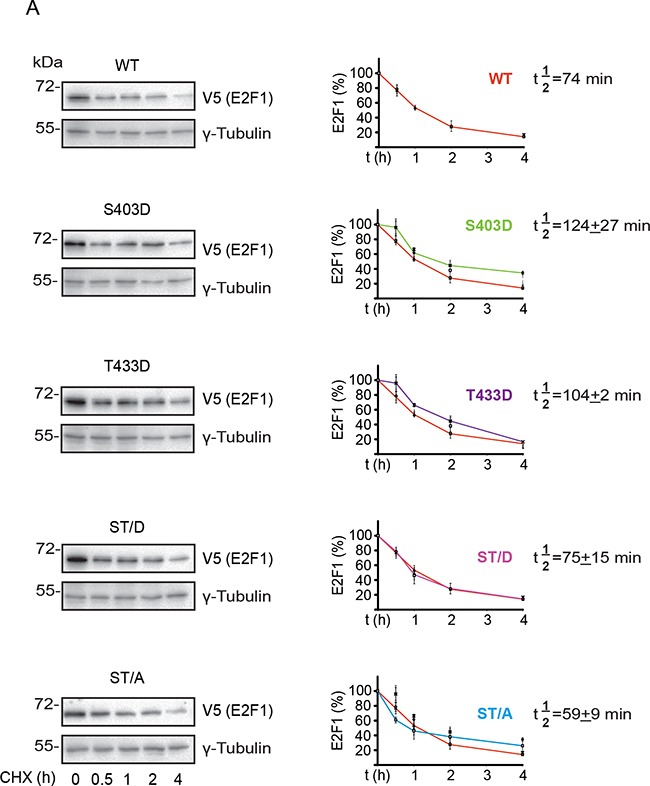

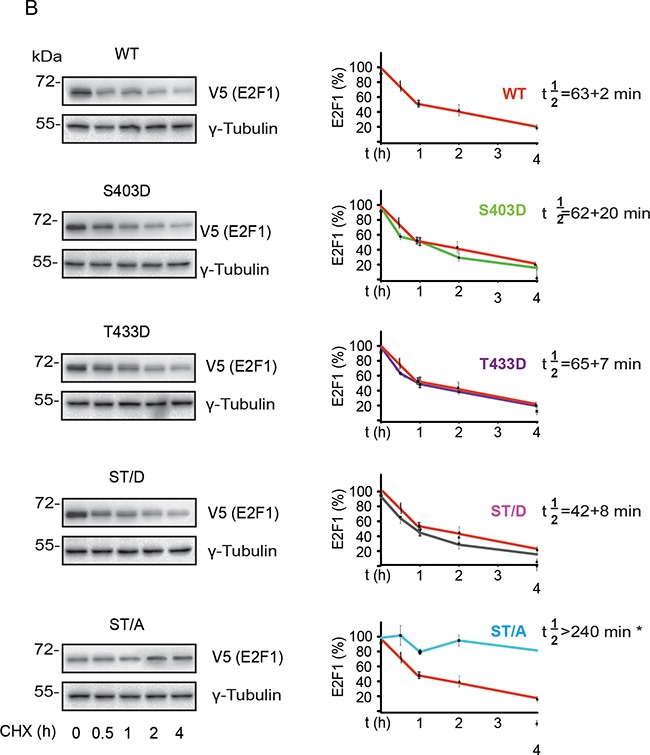
S403 and T433 regulate E2F1 protein half-life in differentiated keratinocytes Undifferentiated keratinocytes were transfected with vectors encoding V5-tagged wild type (WT) or the indicated E2F1 mutant proteins. Twenty-four hours after transfection, the culture medium was replaced with Low Ca^2+^ (panel **A**) or High Ca^2+^. Undifferentiated keratinocytes were transfected with vectors encoding V5-tagged wild type (WT) or the indicated E2F1 mutant proteins. Twenty-four hours after transfection, the culture medium was replaced with High Ca^2+^ (panel **B**) medium, followed by addition of cycloheximide (100 μg/ml, final). Cell lysates were prepared from replicate samples at the indicated times thereafter, and analyzed by immunoblot using anti-V5 antibodies, to detect E2F1, or γ-tubulin, used as loading control. Representative blots for each E2F1 protein are shown. The graphs represent the densitometric quantification of E2F1 protein levels (mean ± S.D., n=3) calculated from experiments conducted on three independent keratinocyte isolates, and are expressed as a percent of levels measured at t=0, set to 100%. Decay curves were used to calculate the apparent half-life of each E2F1 protein (t½), showed on its corresponding graph. For each plot, the decay curve for wild type E2F1 is shown in red, for comparison. The asterisks indicate P<0.05 relative to t½ of wild type E2F1 (ANOVA).

To better understand how phosphorylation might modulate E2F1 turnover, we measured the apparent half-life (t½) of exogenously expressed wild type, S403 and T433 mutants in undifferentiated keratinocytes. The t½ of wild type E2F1 was about 74 min, and was not significantly different from the t½ of the S403D, T433D, ST/D and ST/A mutants (Figure 5A). Similar experiments using differentiated keratinocytes revealed a ~16%-47% decrease in apparent t½ of wild type, S403D, T433D and ST/D mutants in response to Ca^2+^, as compared to their t½ in undifferentiated cells. In stark contrast, E2F1 ST/A was remarkably stable, with a t½>240 min, which is at least 4-fold greater than the other E2F1 proteins examined (Figure 5B). Thus, S403 and T433 phosphorylation appears to be involved in E2F1 turnover in keratinocytes, but only upon differentiation of these cells.

### Link between E2F1 S403/T433 phosphorylation and ubiquitylation

**Figure 6:**
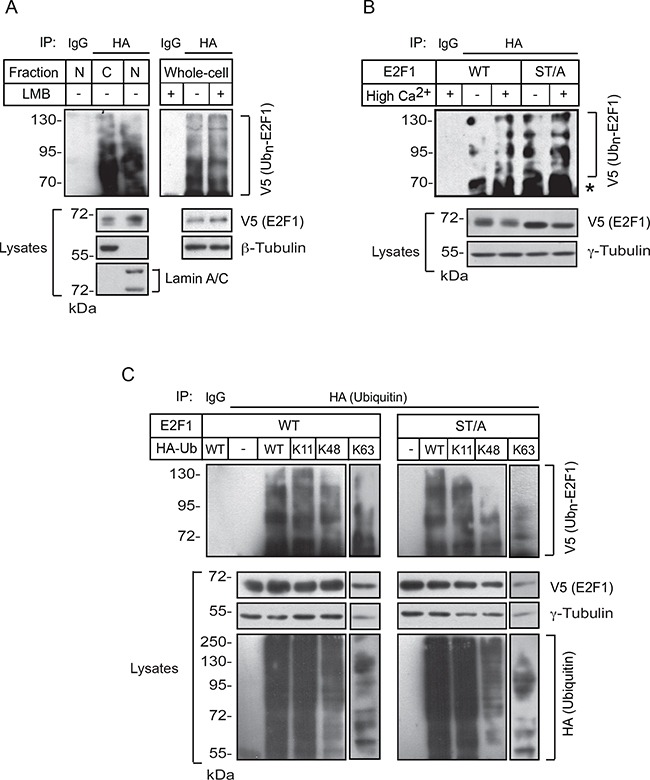
E2F1 ubiquitylation in keratinocytes **A**. Undifferentiated keratinocytes were transfected with vectors encoding wild-type V5-tagged E2F1 and HA-tagged ubiquitin. Twenty-four hours later, MG132 (10 μM, final) was added to the culture medium, followed 3 h later by leptomycin B (LMB, 10 ng/ml, final) or ethanol (vehicle). Cultures were incubated in the presence of both drugs for 30 min, at which time the Ca^2+^ concentration was adjusted to 1.0 mM. Lysates containing whole-cell, nuclear (N) or cytoplasmic (C) fractions were prepared 2.5 h later, HA-ubiquitin immunecomplexes were isolated, resolved by denaturing gel electrophoresis, and analyzed with anti-V5 antibodies to detect ubiquitylated V5-tagged E2F1. Antibodies against β-tubulin or lamin A/C were used to verify the purity of the fractionated extracts and as loading controls. **B**. Undifferentiated keratinocytes were transfected with vectors encoding HA-tagged ubiquitin and V5-tagged wild type or ST/A E2F1, cultured in Low (−) or High (+) Ca^2+^ medium, as indicated, and treated with MG132 as in panel (A). HA or unrelated IgG immunecomplexes were isolated from whole-cell lysates and analyzed by immunoblot with anti-V5 antibodies. The abundance of exogenous E2F1 proteins in the lysates was analyzed by immunoblot, using γ-tubulin as loading control. The asterisk indicates the IgG heavy chain. **C**. Keratinocytes cultured in Low Ca^2+^ medium were transfected with vectors encoding the indicated V5-tagged E2F1 and HA-tagged ubiquitin proteins. Twenty-four hours after transfection, cells were incubated in the presence of MG132 (10 μM, final) for 6 h. Cell lysates were prepared and HA or unrelated IgG immunecomplexes were isolated and analyzed by immunoblot with anti-V5 antibodies. The abundance of exogenous E2F1 and HA-ubiquitin-containing proteins in the lysates was analyzed by immunoblot, using γ -tubulin as loading control. Ub_n_-E2F1 indicates ubiquitylated V5-tagged E2F1 proteins. All results shown are representative of three replicate experiments conducted with independent cell isolates.

E2F1 undergoes proteasomal degradation following ubiquitylation, and increases in its stability have been associated with decreased ubiquitylation [14]. Therefore, we began to characterize the patterns of E2F1 ubiquitylation in keratinocytes. We transfected cells with plasmids encoding wild type V5-tagged E2F1 together with hemagglutinin (HA)-tagged ubiquitin, and isolated nuclear and cytoplasmic fractions following a 24-h interval of culture in High-Ca^2+^ medium in the presence of the proteasomal inhibitor MG132, to prevent E2F1 degradation. HA-immunecomplexes were isolated from these fractions and the presence of V5-tagged E2F1 species was assessed. We were able to detect ubiquitylated E2F1 in both nuclear and cytoplasmic fractions. Further, ubiquitylated E2F1 was also present in whole-cell lysates from keratinocytes treated with leptomycin B, suggesting that ubiquitylation of E2F1 in differentiated keratinocytes does not require nucleocytoplasmic shuttling (Figure 6A). Similarly, we observed the presence of ubiquitylated E2F1 KTR and K185R mutants, which lack lysine residues needed for ubiquitylation in other cell types, irrespective of the differentiation status of the keratinocytes (Supplementary Figure S3). These observations indicate that several of the fourteen lysines present in human E2F1 are ubiquitylated in these cells, and that K117, K120, K125 and K185 are not essential for E2F1 ubiquitylation during keratinocyte differentiation.

We also investigated the role of S403 and T433 in E2F1 ubiquitylation. For these experiments, we transfected keratinocytes with plasmids encoding V5-tagged wild type or ST/A E2F1, together with HA-tagged ubiquitin, and again isolated HA-ubiquitin immunoprecipitates from undifferentiated and from differentiated cells. We observed the presence of multiple ubiquitylated wild type and ST/A E2F1 species in lysates from both undifferentiated and differentiated keratinocytes (Figure 6B), indicating that substitution of S403 and T433 with non-phosphorylatable alanine did not abrogate the overall capacity of E2F1 to serve as a substrate for ubiquitylation.

**Figure 7:**
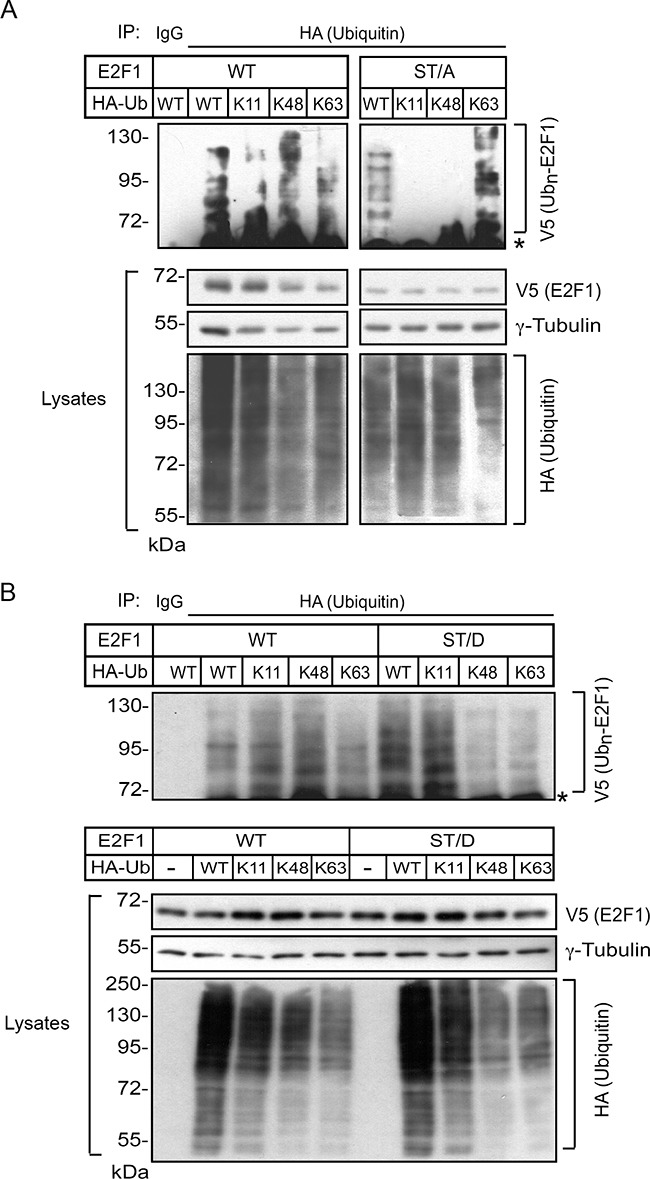
S403 and T433 regulate K11- and K48-linkage ubiquitylation of E2F1 in differentiated keratinocytes Keratinocytes cultured in Low Ca^2+^ medium were transfected with vectors encoding wild type or ST/A V5-tagged E2F1 (Panel **A**), wild type or ST/D V5-tagged E2F1 (Panel **B**) together with plasmids encoding the indicated HA-tagged ubiquitin proteins. After 4 h, the culture medium was replaced with High Ca^2+^ medium, and cells were cultured for 18 h, followed by incubation in the presence of MG132 (10 μM, final) for 6 h. Cell lysates were prepared and HA immunecomplexes were isolated and analyzed by immunoblot with anti-V5 antibodies. The abundance of exogenous E2F1 and HA-ubiquitin-containing proteins in the lysates was analyzed by immunoblot, using γ-tubulin as loading control. Ub_n_-E2F1 signifies ubiquitylated V5-tagged E2F1 proteins, and the asterisk shows the position of the IgG heavy chain. All results shown are representative of at least three replicate experiments conducted with independent cell isolates.

Proteins can have a single ubiquitin added to one or multiple lysine residues, resulting, respectively in mono- or multi-ubiquitylation [20]. In addition, several types of polyubiquitin chains can be added to substrate proteins, depending on which of the seven lysines contained in ubiquitin are utilized for linkage. The resulting modifications can have marked structural and functional consequences. For example, whereas K11 and K48 linkages direct ubiquitylated substrates to proteasomal degradation, ubiquitylation via K63 linkages can modulate the activity of proteins involved in signal transduction and DNA damage, rather than degradation [20]. Therefore, we examined the possibility that S403 and T433 may modulate the type of ubiquitin linkages on E2F1. To this end, we first co-expressed in undifferentiated keratinocytes wild type or ST/A E2F1, together with HA-tagged wild-type ubiquitin or ubiquitin mutants in which all lysines were replaced by arginines, with the exception of K11, K48 or K63. These ubiquitin mutants enhance the formation of polyubiquitin chains containing, respectively, K11, K48 or K63 linkages [21]. We then isolated HA immune complexes and probed them for the presence of V5-tagged E2F1 proteins. In HA immunoprecipitates isolated from undifferentiated keratinocyte lysates, we observed ubiquitylation associated with K11, K48 and K63 linkages in both wild type and ST/A E2F1 (Figure 6C). This indicates that E2F1 polyubiquitylation involving K11, K48 and K63 linkages in undifferentiated keratinocytes does not require S403 and T433. Similar experiments were conducted with lysates from differentiated keratinocytes, but with different outcomes. Although we readily observed the presence of K11 and K48 ubiquitin-modified wild type E2F1, the abundance of K63-linked ubiquitylation appeared to be proportionally lower (Figure 7A and 7B). Notably, E2F1 ST/A ubiquitylation through K63 linkages was readily detected, but little or no detectable K11- and K48-associated modifications were observed (Figure 7A). To further explore a potential role of S403 and T433 phosphorylation in E2F1 ubiquitylation in differentiated cells, we conducted analogous experiments with the pseudophosphorylated mutant E2F1 ST/D, and observed ubiquitylation in the presence of wild type, K11, K48 and K63 ubiquitin, similar to wild type E2F1 (Figure 7B). Together, these data indicate that, upon keratinocyte differentiation, E2F1 can be polyubiquitylated via K11, K48 and K63 linkages. However, the addition of ubiquitin via K11 and K48 linkage might require phosphorylation of S403 and T433 specifically in differentiated keratinocytes. In the absence of S403 and T433 phosphorylation, K63-, but little or no K11- or K48-linked polyubiquitylation is detected in these cells.

### Role of Cdh1 in E2F1 degradation upon induction of keratinocyte differentiation

**Figure 8:**
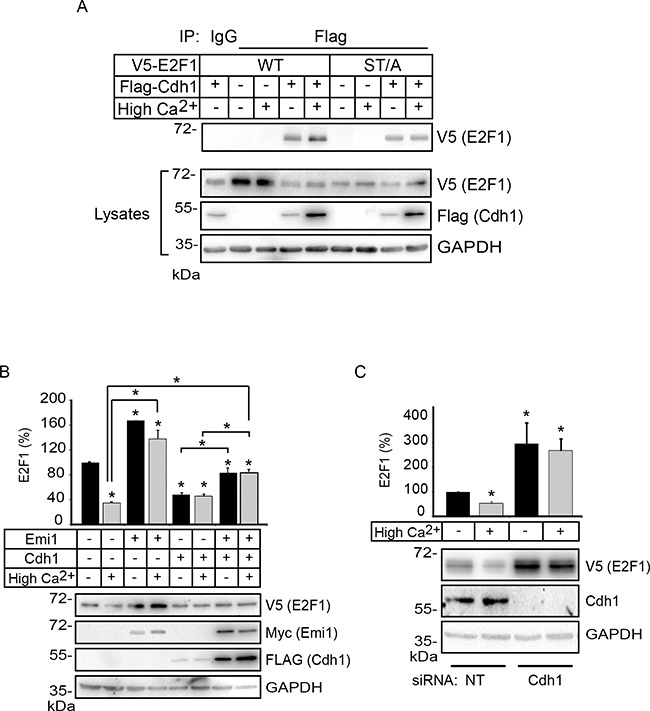
Cdh1 interacts with E2F1 and promotes its degradation in differentiating keratinocytes **A**. Keratinocytes cultured in Low Ca^2+^ medium were transfected with vectors encoding FLAG-tagged Cdh1 and V5-tagged E2F1. After 4 h, the cells were cultured in Low (−) or High Ca^2+^ (+) medium for 24 h. Cell lysates were prepared and subjected to immunoprecipitation with anti-FLAG antibodies. The immune complexes were analyzed by immunoblot with anti-V5 antibodies, to detect E2F1. Samples of the lysates were also analyzed by immunoblot with the indicated antibodies. **B**. Keratinocytes were transfected with vectors encoding V5-tagged E2F1, in the presence or absence of vectors encoding FLAG-tagged Cdh1 and myc-tagged hEmi1, as indicated. Twenty-four hours later, the cells were treated with cycloheximide (100 μg/ml, final) and the culture medium was replaced 30 min later with Low Ca^2+^ (−) or High Ca^2+^ medium. Keratinocyte lysates were prepared 3 h later and analyzed by immunoblot with the indicated antibodies. The histograms represent quantification of E2F1 proteins (mean + SEM, n=3), relative to E2F1 abundance in Low Ca^2+^ medium in the absence of exogenous Cdh1 and hEmi1, which is set at 100%. The asterisks indicate P<0.05 relative to E2F1 levels in cells cultured in Low Ca^2+^ medium without other exogenous proteins, except where otherwise indicated (ANOVA). **C**. Undifferentiated keratinocytes were sequentially transfected with control, non-targeting (NT) or Cdh1-targeting siRNAs, followed by transfection with vectors encoding V5-tagged E2F1, as described in Materials and Methods. Cells were treated with CHX and harvested for protein analysis as described in (B). The histograms represent E2F1 levels (mean + SEM, n=3), relative to those in cells cultured in Low Ca^2+^ medium in the presence of NT siRNA, which is set at 100%. Asterisks indicate P<0.05 relative to E2F1 levels in cells cultured in Low Ca^2+^ medium (ANOVA).

E2F1 is the target of multiple ubiquitin E3 ligases, which regulate its turnover. For example, the anaphase promoting complex/cyclosome E3 ligase activated by Cdc20 (APC/C^Cdc20^) and SCF^Skp2^, respectively mediate E2F1 degradation during prometaphase and the S/G2 phases of the cell cycle [22, 23]. Recently, activation of APC/C by Cdh1 was shown to mediate E2F1 degradation via K11 linkage-specific ubiquitylation in M and early G1 phases, times during which Cdc20 is not present [24]. Significantly, induction of differentiation in human keratinocytes is accompanied by downregulation of *CDC20* and *SKP2* expression, with concomitant upregulation of *FZR1* transcripts, which encode Cdh1 [25]. Thus, we reasoned that Cdh1 might be a reasonable candidate to participate in E2F1 degradation during keratinocyte differentiation. These considerations prompted us to first investigate whether Cdh1 interacts with E2F1 in keratinocytes. To this end, we exogenously expressed wild type V5-tagged E2F1 together with FLAG-tagged Cdh1 in undifferentiated keratinocytes, or in cells that were induced to differentiate by culture in High Ca^2+^ for 24 h. We were able to detect E2F1 in Cdh1 immune complexes in cells cultured both in Low and High Ca^2+^ medium (Figure 8A). We conducted similar experiments to investigate if the lower levels of E2F1 ST/A K11-linked ubiquitylation in differentiated keratinocytes were associated with a decreased ability of this mutant protein to bind Cdh1-containing complexes. We were able to detect E2F1 ST/A in Cdh1 immunoprecipitates isolated from both undifferentiated and differentiated keratinocytes. Thus, although S403 and T433 are dispensable for Cdh1 association with E2F1, they appear to be functionally limiting for K11-linked polyubiquitylation and susceptibility to degradation specifically in differentiated cells (Figure 8A).

To determine the functional significance of the E2F1-Cdh1 interactions, we next investigated the consequences on the abundance of wild type E2F1 following inhibition of Cdh1 activity. To this end, we expressed in keratinocytes the F-box protein hEmi1, which can bind to Cdh1 and interfere with its ability to activate APC/C, thereby stabilizing its substrates [26]. For these experiments, we analyzed changes in the abundance of exogenous V5-tagged E2F1 in response to hEmi1, as this approach allowed us to better detect E2F1, while reflecting in parallel changes in endogenous E2F1. Exogenous expression of hEmi1 induced a slight increase in the abundance of E2F1 in an asynchronous population of undifferentiated keratinocytes (Figure 8B). Notably, hEmi1 prevented the decrease in E2F1 levels associated with differentiation, and E2F1 levels in cells cultured in High Ca^2+^ medium were indistinguishable from those in undifferentiated keratinocytes (Figure 8B). hEmi1 was also able to interfere with the reduction in E2F1 levels in both undifferentiated and differentiated keratinocytes observed upon overexpression of Cdh1. To complement these studies, we investigated the effect of Cdh1 silencing on the regulation of E2F1 using RNA interference. We observed a three-fold increase in E2F1 levels in undifferentiated keratinocytes treated with Cdh1-targeting siRNA, which was unaffected by induction of differentiation following Ca^2+^ treatment (Figure 8C). Together, our observations are consistent with the notion that Cdh1 is a major contributor to E2F1 degradation during epidermal keratinocyte differentiation.

## DISCUSSION

One of the most important questions in the biology of E2F1 is how it is differentially regulated in response to a wide and complex variety of physiological stimuli, such as differentiation signals. E2F1 is downregulated to allow proper entry into quiescence and expression of differentiation markers in the epidermis [12, 13]. Our studies now describe a multifactorial mechanism of E2F1 degradation during keratinocyte differentiation that involves coordinate changes in subcellular localization and ubiquitylation patterns, and in which S403 and T433 play key modulatory roles.

E2F1 has a relatively rapid turnover, but several residues located in the N-terminal half of this protein participate in its stabilization and escape from proteasomal degradation in response to DNA damage. Specifically, post-translational modifications of S31, K107, K120, K125, K185, R111 and R113 collectively increase E2F1 stability under these circumstances [1]. In keratinocytes, E2F1 also participates in repair of DNA photodamage induced by UV radiation through various mechanisms, including association with hHR23 proteins involved in nuclear excision repair [9]. Under these conditions, hHR23 proteins bind to E2F1 and protect it from proteasomal degradation. Significantly, E2F1 ST/A and E2F1 deletion mutants lacking S403 and T433 can bind to hHR23A and become stabilized upon UV irradiation in undifferentiated keratinocytes ([9]; Singh and Dagnino, unpublished observations), indicating that the regulation of E2F1 turnover upon DNA damage and differentiation proceed through distinct mechanisms. Significantly, none of the residues involved in E2F1 stabilization following DNA damage are required for E2F1 downregulation during keratinocyte differentiation, which instead depends on the integrity of S403 and T433. This emphasizes the clear distinction between mechanisms activated in response to physiological and pathological signals. E2F1 undergoes nucleocytoplasmic shuttling in undifferentiated keratinocytes and other cell types, although the balance of these movements favours a predominantly nuclear localization at steady-state [19]. Upon induction of differentiation, E2F1 is excluded from the nucleus and retained in the cytoplasm in keratinocytes and hippocampal neurons [12, 27]. Based on the ability of wild type and phosphomimetic E2F1 ST/D, but not E2F1 ST/A, to respond to Ca^2+^, we propose that such nucleocytoplasmic translocation is potentially dependent on phosphorylation of S403 and T433. Our data also show that nuclear export does not require residues involved in E2F1 responses to DNA damage. However, nuclear export is not sufficient for E2F1 degradation during differentiation, as evidenced by the stability of the constitutively cytoplasmic E2F1 mutant ST/A-NES. Thus, multiple factors are necessary for efficient degradation of E2F1 in response to differentiation signals in epidermal cells.

E2F1 stability and transcriptional activity can be modulated through various types of ubiquitin linkages. In differentiated and in undifferentiated keratinocytes, we observed evidence of K11- K48- and K63-linked polyubiquitylation, in agreement with similar findings reported for various tumour cell lines [24, 28, 29]. Polyubiquitylation via K63 linkages does not appear to target E2F1 for proteasomal degradation, but rather decreases its transcriptional activity [29]. During the G1/S transition and in S phase, K63-linked polyubiquitin chains are removed from E2F1 by the deubiquitylating enzyme UCH37. This modification increases E2F1 transcriptional activity, resulting in increased expression of target genes that promote cell proliferation [29]. Similarly, following DNA damage, removal of K63-linked polyubiquitin chains activates transcription of pro-apoptotic genes [29]. Our data show that K63-linked ubiquitin modifications are present in keratinocytes, irrespective of their differentiation status. Given that this modification was present in wild type, ST/A and ST/D E2F1 proteins, our data indicate that S403 and T433 do not determine the susceptibility of E2F1 for K63-linked polyubiquitylation. An important area for future research will be to determine the role of K63-linked polyubiquitylation of E2F1 during keratinocyte differentiation and entry into quiescence.

E2F1 ubiquitylation via K11 and K48 linkages is required for proteasomal degradation. In addition, post-translational modifications on E2F1 that subsequently alter its ubiquitylation patterns regulate both its cell cycle phase-specific degradation and its stabilization during DNA damage [1, 30]. E2F1 degradation at the onset of S/G2 is achieved through the action of several E3 ubiquitin ligases. Specifically, SCF^Skp2^ and Cdc20-activated APC/C (APC/C^Cdc20^) promote E2F1 turnover during S and M phase, respectively [22, 31]. This process requires an intact C-terminal domain in E2F1, and in particular the region encompassing amino acid residues 363-452 [32, 33]. Although our experiments did not directly test the role of S403 and T433, these two residues may not be indispensable for cell cycle-related regulation of E2F1 abundance, given that the t½ we observed for the mutants E2F1 ST/A and ST/D were fairly similar to the t½ of the wild type protein in undifferentiated, exponentially proliferating keratinocytes. In contrast, these two residues appear to be critical components of the E2F1 degron implicated in E2F1 proteolysis during keratinocyte differentiation.

The APC/C complex is a multisubunit E3 ligase composed of 15 different proteins [34, 35]. Its catalytic module is composed of APC2 and APC11, and recruits E2 ligases, such as UBE2S, which assembles K11-linked polyubiquitin chains on client proteins. APC/C requires association with one of its coactivators, Cdc20 or Cdh1, to induce substrate ubiquitylation. Cdc20 and Cdh1 recognize and specifically bind myriad substrates, delivering them to the APC/C complex [34, 35]. In exponentially proliferating cells, E2F1 is targeted for degradation by APC/C^Cdc20^ during prometaphase, and by APC/C^Cdh1^ in the late M and early G1 phases [22, 24]. APC/C^Cdh1^ also regulates the stability of E2F3, E2F7 and E2F8 during normal cell cycle progression in a variety of cell types [36, 37].

The regulatory role of Cdh1 goes well beyond cell cycle progression. In addition to our data implicating Cdh1 in E2F1 degradation during keratinocyte differentiation, Cdh1 is also required for induction and maintenance of quiescence in other cell types, cooperating with retinoblastoma family proteins and cyclin-dependent kinase inhibitors to maintain cells in the G0/G1 phase [38]. Cdh1 is also necessary to prevent post-mitotic neuron re-entry into the cell cycle, thus playing key roles in neuronal development [38].

Multiple domains in Cdh1 contribute to its association with several subunits in the APC/C complex, and these multivalent interactions determine distinct functional outputs [39]. The ubiquitylation of a substrate not only depends on its binding to Cdh1, but also on its relative position and proximity to the K11-linking machinery [39]. We determined that both wild type and ST/A E2F1 bind to Cdh1. However, whereas K11 polyubiquitin modifications are readily detected in wild type E2F1, they are barely present in E2F1 ST/A. It is possible that phosphorylation of S403 and T433 may interfere with optimal positioning of E2F1 for subsequent polyubiquitylation mediated by the APC/C^Cdh1^ complex. Alternatively, Cdh1 and E2F1 ST/A may associate outside the context of APC/C. In this regard, it has been established that Cdh1 also fulfills APC/C-independent functions, including promotion of axonal growth in differentiated neurons, inhibition of the E2 ligase WWP2 with stabilization of PTEN and activation of the Smurf1 ubiquitin ligase to interfere with osteoblast differentiation [40–42]. E2F1 ST/A also shows deficits in K48-linked polyubiquitylation, suggesting the involvement of additional mechanisms that regulate E2F1 proteasomal degradation. An important area for future research will be to determine the effect that phosphorylation of S403 and/or T433 has on E2F1 conformation and its interactions with APC/C^Cdh1^ and other ubiquitylation complexes. In our studies, hEmi1-induced inhibition or siRNA-mediated silencing of Cdh1 interfered with E2F1 downregulation in differentiating keratinocytes. Given that the latter process is necessary for normal keratinocyte differentiation, loss of *Fzr*, which encodes Cdh1, would be expected to interfere with normal epidermal morphogenesis *in vivo*. The generation and analysis of mice with epidermis-restricted *Fzr* inactivation will provide future answers to this important question.

In summary, our results provide mechanistic insight into a series of complex, differentiation-specific molecular mechanisms that regulate E2F1 during keratinocyte maturation via multiple events. These events include nucleocytoplasmic transport and changes in ubiquitylation patterns specifically orchestrated through S403 and T433, and which differ from other mechanisms that regulate E2F1 turnover in undifferentiated cells.

## MATERIALS AND METHODS

### Cell culture, transfection and treatments

All experiments using mice were approved by the Animal Care Committee of the University of Western Ontario (Protocol No. 2015-021), in accordance with regulations from the Canadian Council on Animal Care. Primary mouse keratinocytes were isolated from skin harvested from 2d-old CD-1 mice. Undifferentiated keratinocytes were cultured in “Low Ca^2+^ medium”, composed of Ca^2+^-free Eagle's minimum essential medium (06-174G, Lonza, Rockland, ME) supplemented with growth additives and 8% fetal bovine serum (FBS) pre-treated with Chelex resin, as described [7, 13]. Keratinocyte differentiation was induced by culture in growth medium supplemented with 1 mM CaCl_2_ (“High-Ca^2+^ medium”) for intervals indicated in individual experiments. Transfections were conducted with polyethyleneimine (PEI), as described [43]. For E2F1 turnover experiments, cycloheximide (CHX, 100 μg/ml, final) was added to keratinocyte cultures 24 h after transfection and, where applicable, 30 min prior to adjusting Ca^2+^ to 1.0 mM; cell lysates were prepared at timed intervals after CHX addition and analyzed by immunoblot. For experiments involving proteasomal inhibition, cells were treated with MG132 (10 μM, final) for 6 h prior to harvesting. Leptomycin B (LMB, 5 ng/ml, final) was added to cultures 24 h after transfection and 6 h prior to cell harvesting. For experiments inducing DNA damage, keratinocytes were transfected, 24 h later they were incubated in the presence or absence of etoposide (150 μM, final), and 8 h after drug addition they were harvested to prepare cell lysates.

### Cdh1 silencing

For experiments using siRNA, cells were transfected with Lipofectamine 2000 and siRNA (100 nM) for 4 h, following the manufacturer's instructions. The medium was replaced with Low Ca^2+^ growth medium. Seventy-two hours after siRNA treatment, keratinocytes were transfected a second time using PEI and plasmids encoding V5-tagged E2F1 and 21 h later, the cells were treated with CHX for 30 min. The culture medium was then replaced with fresh Low or High Ca^2+^ medium supplemented with CHX, and the cultures were processed 2.5 h later to obtain protein lysates. The control siRNA used was the *Silencer* Negative Control #1 (AM4611, ThermoFisher Scientific, Rockford, IL). The sequences of the Cdh1-targeting siRNAs (si*Fzr1* siGENOME SMARTpool (M-065289-00, Dharmacon, GE Healthcare, Lafayette, CO) were: 5′-GCAAUGACGUGUCCCCAUA-3′, 5′-UGGAAC CACUCUAGUCUAA-3′, 5′-GGUCCAAGCACGC CAAUGA-3′, and 5′-CGCCAGAGCUUCAGGACGA-3′.

### Reagents and antibodies

Reagents and their suppliers were as follows: Chelex 100 resin from BioRad (142-2832; Mississauga, ON, Canada), cholera toxin (100) from List Biological (Campbell, CA, USA), leptomycin B (ALX-380-100) from Enzo Life Sciences (Plymouth, PA), PEI (25 kDa linear, Cat. No. 23966) from Polysciences (Warrington, PA). All other reagents were purchased from Sigma (St. Louis, MO, USA). The antibodies used were: anti-V5 (R960-25; Invitrogen, Carlsbad, CA), anti-HA (Y-11) from Santa Cruz (Santa Cruz, CA), anti-glyceraldehyde 3-phosphodehydrogenase (GAPDH; ADI CSA 335) from Enzo Life Sciences (Farmingdale, NY), anti-γ-tubulin (T6557) from Sigma (St. Louis, MO); anti-Cdh1 (DH01-DCS-266, ab3242, Abcam, Cambridge, UK), and anti-c-myc (SC-40, Santa Cruz). Horseradish peroxidase-conjugated goat anti-mouse (115–035-146) and anti-rabbit (111-035-144) antibodies were from Jackson Immuno Research Laboratories (West Grove, PA). Alexa Fluor®-conjugated goat anti-rabbit and goat anti-mouse IgG were from Molecular Probes/Thermo Fisher Scientific (Eugene, OR). λ phosphatase (PO753S) was from New England Biolabs (Ipswich, MI).

### Plasmids

The plasmids encoding V5-tagged wild type human E2F1, E2F1 S403A, T433A and S403A/T433A mutants have been described [12, 13]. All other mutant E2F1 plasmids were generated by site-directed mutagenesis and PCR amplification, using the vector encoding V5-tagged wild type E2F1 as a template. All vectors were verified by dideoxy sequencing. The following plasmids were obtained from Addgene (Cambridge MA): pRK5-HA-Ubiquitin-K11 (#22901), -K48 (#17605), -K63 (#17606) and pRK5-HA-Ubiquitin-WT (#17608), originally deposited by Drs. T. Dawson and C. Pickart, and pCMV2-FLAG-tagged UBC13 (#12460), deposited by Dr. D. Zhang. Mammalian expression vectors encoding hEmi1 and Cdh1 were generous gifts from Dr. P. K. Jackson (Stanford University School of Medicine), and have been previously described [36].

### Immunoblot analysis and immunoprecipitation

Cell lysates were prepared with a modified RIPA-lysis buffer (50 mM Tris-HCl pH 7.4, 150 mM NaCl, 1% NP-40, 0.5% sodium deoxycholate, 0.1% sodium dodecylsulphate (SDS), 1 mM Na_3_VO_4_, 5 mM NaF, 1 mM phenylmethylsulfonyl fluoride, 10 mg/ml each aprotinin, leupeptin and pepstatin). In experiments assessing E2F1 ubiquitylation, the lysis buffer was supplemented with 25 mM N-ethylmaleimide (NEM). Proteins in lysates (30 μg/sample) were resolved by denaturing polyacrylamide gel electrophoresis, transferred to polyvinyledene fluoride membranes, which were probed with antibodies indicated in individual experiments. For immunoprecipitation assays, cell lysates (1.5 mg protein/sample) were incubated with antibodies indicated in individual experiments (3 μg/sample) for 16 h at 4°C. The immune complexes were incubated with A/G magnetic beads (88802, Pierce Biotechnology/Thermo Fisher Scientific, Rockford, IL) for 1 h at 22°C, followed by 5 washes with PBS containing 0.05% Tween-20, and elution in 1x Laemmli sample buffer for 10 min at 25°C. Immune complexes were resolved and analysed by immunoblot, as described above. The results shown in figures are representative of 3-5 experiments, each conducted with independent cell isolates.

### Indirect immunofluorescence microscopy

Unless otherwise indicated, keratinocytes were processed for microscopy 24 h after transfection, as described [12]. The fraction of cells exhibiting E2F proteins with nuclear or cytoplasmic distribution was determined from ≥100 transfected cells per experiment. Images were obtained with a Leica DMIRBE fluorescence microscope equipped with an Orca-ER digital camera (Hamamatsu Photonics, Hamamatsu City, Japan), using Volocity 6.1.1 software (Improvision-PerkinElmer, Waltham, MA). All photomicrographs shown are representative of 3-5 experiments conducted on duplicate samples, and using independent cell isolates.

### Statistical analysis

Data are expressed as mean + standard error, and statistical analyses were conducted, as appropriate, with one-way or two-way analysis of variance (ANOVA), followed by Newman-Keuls or Bonferroni post-hoc test, respectively. Statistical analyses were conducted with GraphPad Prism Version 7 software, and significance was set at P<0.05.

## SUPPLEMENTARY FIGURES



## Abbreviations

APC/C: anaphase promoting complex/cyclosome
CHX: cycloheximide
FBS: fetal bovine serum
GAPDH: glyceraldehyde 3-phosphodehydrogenase
HA: hemagglutinin
LMB: leptomycin B
NEM: N-ethylmaleimide
NES: nuclear export sequence
PBS: phosphate-buffered saline
SDS: sodium dodecylsulfate
t½: half-life.
