# Supplementary material for: CDH1 regulates E2F1 degradation in response to differentiation signals in keratinocytes
Source: Oncotarget. 2016 Nov 26;8(3):4977–93. doi: 10.18632/oncotarget.13636 (PMC5354885; doi:10.18632/oncotarget.13636)
Supplement: Supplementary file 1 [file oncotarget-08-4977-s001.pdf]

CDH1 regulates E2F1 degradation in response to differentiation signals in keratinocytes

SUPPLEMENTARY FIGURES

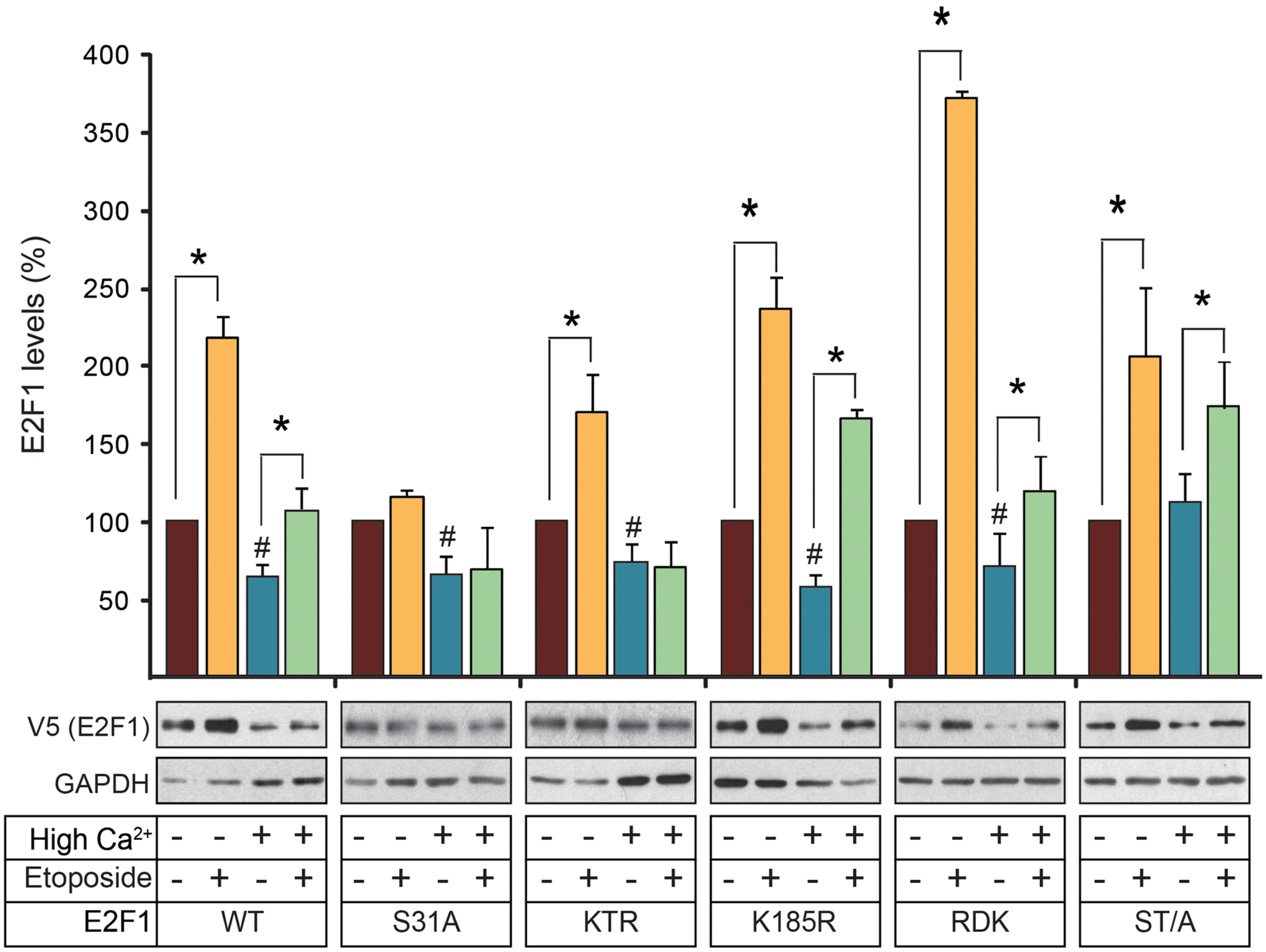

Supplementary Figure 1: Changes in E2F1 levels in response to etoposide. Vectors encoding V5-tagged wild type (WT) or the indicated E2F1 mutants were transfected in undifferentiated keratinocytes. Four hours after transfection, the cells were cultured in Low (-) or High Ca^{2+} (+) medium, and 24 h later, they were incubated in the presence or absence of etoposide (150 mM, final). Cell lysates were prepared 8 h later and analyzed by immunoblot, using anti-V5 antibodies, or GAPDH, as loading control. The histograms represent normalized densitometric quantification of each E2F1 protein (mean + SEM, n=3), and are expressed as the percentage of a given E2F1 form relative to its abundance in Low Ca^{2+} medium, which is set at 100%. The asterisks indicate $P<0.05$, and # indicates $P<0.05$ relative to the corresponding E2F1 mutant protein levels in cells cultured in Low Ca^{2+} medium in the absence of etoposide (ANOVA).

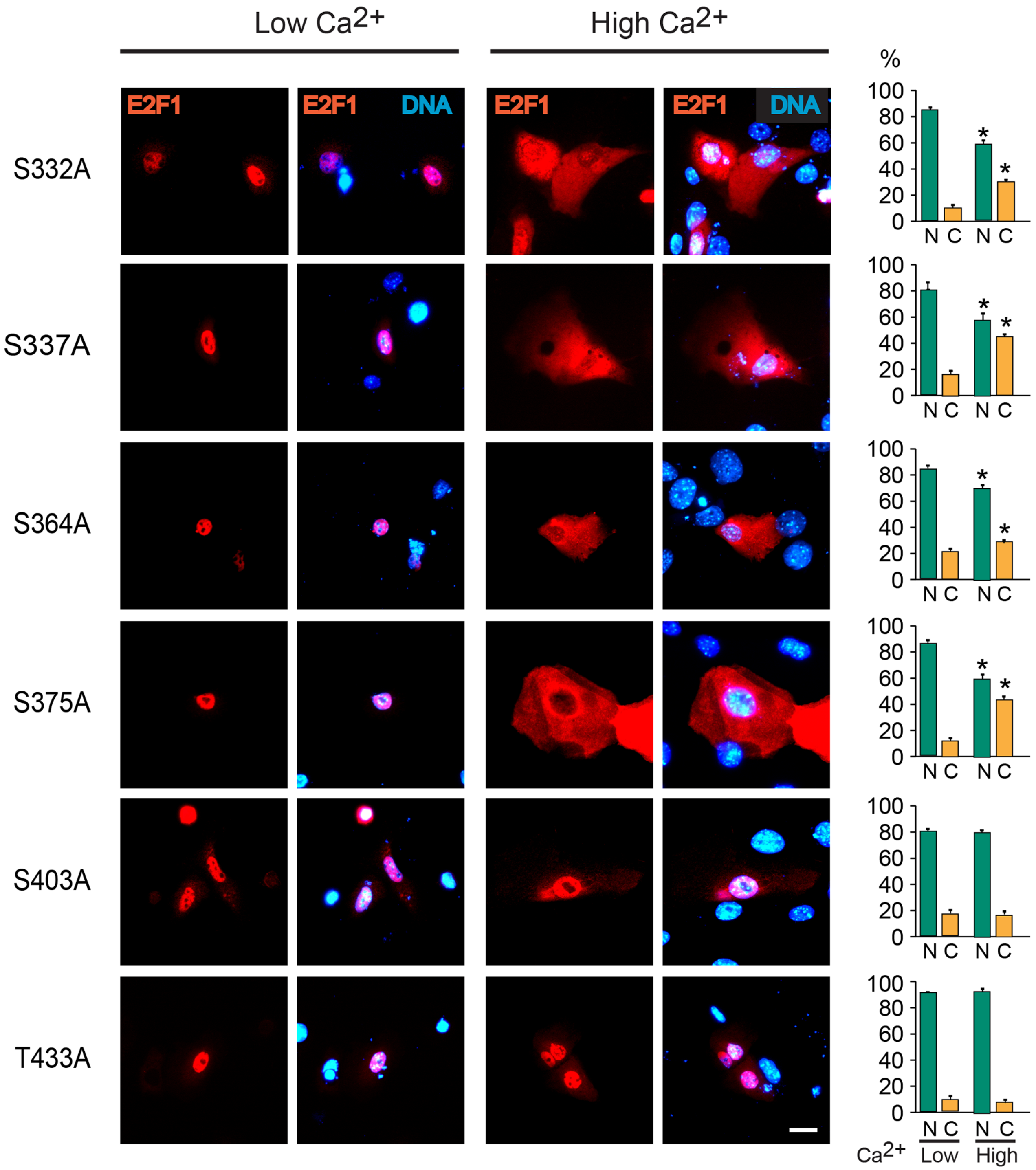

Supplementary Figure S2: Nuclear export of E2F1 mutant proteins during keratinocyte differentiation. Undifferentiated keratinocytes were transfected with vectors encoding the indicated V5-tagged E2F1 mutant proteins. Four hours after transfection, the cells were cultured in Low Ca^{2+} medium, and were processed for immunofluorescence microscopy 24 h later, using anti-V5 antibodies. DNA was visualized with Hoechst 33342. The values in the histograms represent the percentage of cells (mean + SEM, n=3) that exhibited nuclear (N) or cytoplasmic (C) E2F1 distribution. The asterisks indicate $P<0.05$ relative to values in the corresponding subcellular compartments in Low Ca^{2+} medium (ANOVA). Bar, 16 µm.

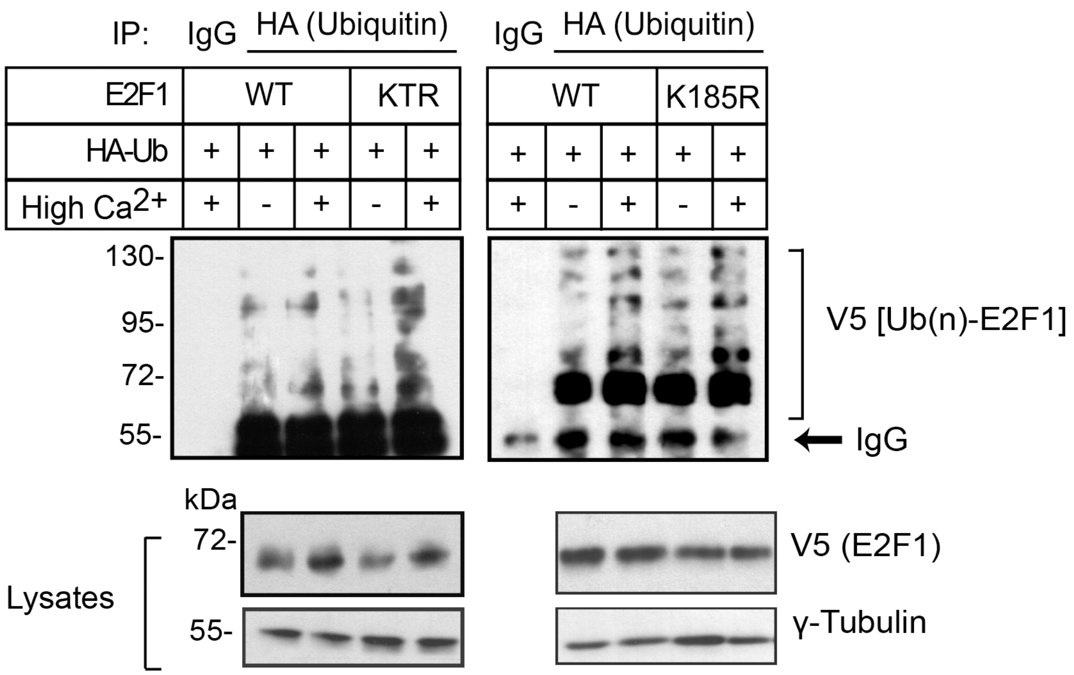

Supplementary Figure S3: Ubiquitylation of E2F1 proteins in keratinocytes. Undifferentiated keratinocytes were transfected with vectors encoding HA-tagged ubiquitin and the indicated V5-tagged E2F1 proteins, cultured in Low (-) or High (+) Ca^{2+} medium, treated with MG132 (10 mM, final) for 3 h, and harvested to prepare whole-cell lysates. HA or unrelated IgG immunoprecipitates were isolated from the lysates and analyzed by immunoblot with anti-V5 antibodies. The abundance of exogenous E2F1 proteins in the lysates was analyzed by immunoblot, using γ-tubulin as loading control.
